# Malarial Hæmaturia

**Published:** 1890-11

**Authors:** H. A. Tutwiler

**Affiliations:** Flatonia, Texas


For Daniel's Texas Medical Journal.

### MALARIAL HÆMATURIA.—HEMORRHAGIC MALARIAL FEVER.—YELLOW DISEASE, &C.

BY H. A. TUTWILER, M. D., FLATONIA, TEXAS.

Read at the West Texas Medical Association Meeting August 8, 1890.

THIS disease, known by each of the above names, is, in our judgment, one of the most important that we are called on to treat throughout the South. From South Carolina to the Rio Grande, there is hardly a county in some part of which it is not prevalent. A disease, not of our cities, in which it is rarely found, but confined to our creek and river bottoms, where the physician has no drug stores to call upon, and few, if any opportunities of calling in medical assistance. Dependent entirely upon his own resources and with the facts staring him in the face, first, that it is one of our most fatal diseases, the mortality ranging from forty to sixty per cent.; and second, that the treatment as adopted by different practitioners is so different, that it is hardly to be wondered at that one hesitates which method to adopt, losing valuable time, or adopts that plan of treatment which the results show not to have been the best. That it is a disease of comparatively recent date is shown by the fact that our medical works make no mention of it. For instance, in the Practice of Medicine by Dr. Samuel Dickson, of South Carolina, there is nothing to be found concerning it; although we know he was one of the foremost practitioners of his time. A man of accurate observation, ripe knowledge and large experience. Even in those of our late medical works to which we have had access, we have found very little on the subject. The causes are essentially malarial, although we have long thought that there must be some additional cause, from the fact that although we have malarial fevers here every year, often of a pernicious type, and with a high rate of mortality, yet we often pass over several years without a single case of this disease. The pathology, as given by those authors whom we have been able to consult, is just about what we might expect from the external appearance,

AN INTENSE BILIOUS CONDITION

of all the viscera, even the blood itself being highly charged with bile. The kidneys much congested. The liver and spleen enlarged and softened, the latter often breaking down at the mere touch of the finger; brain in its natural condition. Dr. Berangen Feraud characterizes the disease "as a pyrexia of variable intensity, accompanied by an intense jaundiced condition of the skin and a discharge of dark colored, almost black urine, with vomiting of bilious matter and sometimes of blood." There is great prostration, a high temperature accompanied with a phenomenally slow pulse, often intermittent. The tongue is dry and covered with a thick, dirty, yellowish white coat. The bowels are almost always constipated." Men are more likely to contract the disease than women, and it is more fatal with the former. My experience also has been that brunettes are far more likely to suffer than blondes. We have never seen a case in a negro, but have heard of several.

The diagnosis is so simple and so plain that it is hardly necessary to do more than glance at it. After exposure to malarial influence for a variable period, the patient is taken with rigors, pain in the region of kidneys, soon followed by a high grade of fever, the jaundiced condition of skin, the prostration, nausea, bilious vomiting, and dark colored urine. It is generally the case that by the time the physician reaches the bedside the condition is so pathognomonic that it would be next to impossible to make a mistake. The chances of an attack as given by Ferand are as follows: "After one year's residence in a malarial region, 5 per cent., after two years 22 per cent., after three years 42 per cent. The danger of a second, third or fourth attack is even greater."

We have known cases in which the patients were prostrated as often as four times, by the disease, in as many years, and finally dying from it.

TREATMENT.

So far as the causes and diagnosis are concerned, we find no ground for dispute among the medical profession, whereas now we find differences of opinion so great that it is impossible to reconcile them. We believe first and foremost that evacuants are more strongly called for, in the begining, than any other remedy, and that without their aid, all our efforts to relieve the bilious condition or to cure the infection will be futile. And our experience has led us to favor calomel for this purpose, above all other remedies. For an adult we rarely begin with less than ten grains and repeat every two hours until the bowels are thoroughly cleansed out. We give it dry on the tongue, allowing the patient to wash it down with a small drink of water. It not only relieves the constipated bowels, and assists in carrying off the surplus bile, but given in this way it acts as a diuretic, and is the only diuretic from which we have ever seen any benefit. If possible, we give the patient a hot bath, and by *hot* we mean just as hot as it can be borne. Repeat it as often as necessary. It stimulates the patient and relieves the kidneys of much hard work. To check the nausea and vomiting, we have found nothing better than hot water, sipped slowly and often, or a hot infusion of peach leaves, made fresh every hour or two, and used in the same manner. Do not make any effort to force the kidneys by using the medicines known as diuretics. During our experience in this county of twenty-four years we never remember to have seen any benefit from their use. Mustard applied to the stomach, wrists, ankles and spine; kept on until considerable counter-irritation is set up without blistering, is often beneficial, but we deprecate the use of blisters. In the few cases that we have known of their being used they did great harm without any compensating benefit. As soon as the bowels are moved, or even before, give quinine with a liberal hand, never mind about weighing it. Give in teaspoonful doses, and repeat it just as rapidly as the patient will bear it, until you get its constitutional effect. Ten or fifteen minutes before each dose of the quinine give a hypodermic injection of morphia. Feraud says it "does good by checking the bilious condition." Whether it does or not, we cannot say, but we do know that it benefits our patient to retain the quinia salt. We remember an almost hopeless case which we treated two years ago. We gave one drachm sulphate of quinia within two hours, together with one quarter grain dose of morphia hypodermically every few minutes [?] until the patient was thoroughly under its influence. He made a good recovery although he was past middle age, and was much broken down from chronic malaria.

Where there is partial heart failure we have several times had good results from the use of caffein. It certainly seems to me to act more quickly, and its action to last longer than any other remedy we have seen tried. As my experience with this disease is almost entirely limited to this county, we wish the remarks to be understood as applying only to this county. The disease here is never, as far as my knowledge goes, seen on our prairies, unless brought from the swamps. Therefore we have made it a rule to strenuously insist on the removal of the patient beyond the danger point; which here will be only a matter of a few miles. Even before he can sit up, haul him out, and my word for it, with ordinary care and nursing, you will find your patient improve for every day and every mile of change. As every patient is more likely to suffer a second or third attack than he was the first, it is best to advise an entire change of climate, and if that is impracticable, at least a change for a few months. There having been but little of this disease here since the introduction of antipyrin, acetanilide, phenacetine, etc., we have had no opportunity of trying them sufficiently to express an opinion as to their merits. And as all my cases have been too far from railroads to procure ice with facility, we cannot say much about it. From what little experience we have had, we have hardly been favorably impressed with it. Nor would we fail to exercise great caution if we should see occasion to use it in the future. We have corresponded during the year with many physicians, both in this and other States in regard to this disease, and although their ideas and opinions differed widely in many respects, yet we only received one communication in which the writer objected to the use of calomel. He claiming that it did no good, but was hurtful, and sometimes the cause of death. Of the same opinion is Dr. Feraud who, in writing of "melanuric bilious fever," as he calls it, says: "After more than three hundred observations, I have become convinced that calomel is an inefficient and dangerous medicine in this disease, and if my ideas were adopted, calomel would *be excluded from its treatment.*" This is the opinion of the chief surgeon of the French navy who was sent to the French Colonies by his government for the express purpose of reporting upon the disease, and who, we believe, remained there a year or more. Such an opinion, from such a source, cannot be treated lightly, however much we may differ with its author. We had some thoughts of giving extracts from the many letters received by us, but this would render the present essay too long for your patience, which has already been sufficiently taxed. If what has been written calls forth opinions from better informed and more capable physicians and results in good to humanity and to our profession it will have accomplished all the writer desires or expects.

---

Dr. Schaffer recommends the use of suppositories cut from a cake of glycerine soap as a substitute for glycerine enemata. The soap is said to contain 30 per cent. or more of glycerine, and the remedy is as effectual in this form, he claims, as it is when used pure.—*New England Med. Monthly.*

